# Involvement of PPARγ in the Anticonvulsant Activity of EP-80317, a Ghrelin Receptor Antagonist

**DOI:** 10.3389/fphar.2017.00676

**Published:** 2017-09-22

**Authors:** Chiara Lucchi, Anna M. Costa, Carmela Giordano, Giulia Curia, Marika Piat, Giuseppina Leo, Jonathan Vinet, Luc Brunel, Jean-Alain Fehrentz, Jean Martinez, Antonio Torsello, Giuseppe Biagini

**Affiliations:** ^1^Laboratory of Experimental Epileptology, Department of Biomedical, Metabolic and Neural Sciences, University of Modena and Reggio Emilia Modena, Italy; ^2^Centre National de la Recherche Scientifique, Max Mousseron Institute of Biomolecules, National School of Chemistry Montpellier, University of Montpellier Montpellier, France; ^3^School of Medicine and Surgery, University of Milano-Bicocca Milan, Italy; ^4^Center for Neuroscience and Neurotechnology, University of Modena and Reggio Emilia Modena, Italy

**Keywords:** 6-Hz corneal stimulation, EP-80317, ghrelin, peroxisome proliferator-activated receptor gamma, pilocarpine, status epilepticus, seizure

## Abstract

Ghrelin, des-acyl ghrelin and other related peptides possess anticonvulsant activities. Although ghrelin and cognate peptides were shown to physiologically regulate only the ghrelin receptor, some of them were pharmacologically proved to activate the peroxisome proliferator-activated receptor gamma (PPARγ) through stimulation of the scavenger receptor CD36 in macrophages. In our study, we challenged the hypothesis that PPARγ could be involved in the anticonvulsant effects of EP-80317, a ghrelin receptor antagonist. For this purpose, we used the PPARγ antagonist GW9662 to evaluate the modulation of EP-80317 anticonvulsant properties in two different models. Firstly, the anticonvulsant effects of EP-80317 were studied in rats treated with pilocarpine to induce *status epilepticus* (SE). Secondly, the anticonvulsant activity of EP-80317 was ascertained in the repeated 6-Hz corneal stimulation model in mice. Behavioral and video electrocorticographic (ECoG) analyses were performed in both models. We also characterized levels of immunoreactivity for PPARγ in the hippocampus of 6-Hz corneally stimulated mice. EP-80317 predictably antagonized seizures in both models. Pretreatment with GW9662 counteracted almost all EP-80317 effects both in mice and rats. Only the effects of EP-80317 on power spectra of ECoGs recorded during repeated 6-Hz corneal stimulation were practically unaffected by GW9662 administration. Moreover, GW9662 alone produced a decrease in the latency of tonic-clonic seizures and accelerated the onset of SE in rats. Finally, in the hippocampus of mice treated with EP-80317 we found increased levels of PPARγ immunoreactivity. Overall, these results support the hypothesis that PPARγ is able to modulate seizures and mediates the anticonvulsant effects of EP-80317.

## Introduction

Ghrelin and des-acyl ghrelin are neuroactive peptides prevalently produced in the stomach by X/A-like cells in rats, or P/D1 cells in humans (Rindi et al., 2002; Kojima, 2005; Chen et al., 2009). They regulate very important physiological functions, such as growth hormone secretion, food intake and metabolism by interacting with hypothalamic neurons (Nakazato et al., 2001). Ghrelin has also been investigated for its possible role in regulating neuronal activity in other brain regions, since its receptor was found to be expressed out of the hypothalamus and especially in the hippocampus (Zigman et al., 2006). Interestingly, ghrelin was demonstrated to play an important role in learning and memory, cognitive functions in which the hippocampus is critically involved (Diano et al., 2006; Carlini et al., 2010; Li et al., 2013). Furthermore, ghrelin was also shown to modulate energy metabolism in areas other than the hypothalamus, as well as to be involved in the rewarding system of the brain (Egecioglu et al., 2010; Skibicka and Dickson, 2011; Skibicka et al., 2011; Perelló and Zigman, 2012). Overall, these evidences delineate a variety of physiological roles for ghrelin in the central nervous system (CNS).

Ghrelin has also been implicated in cerebral diseases. Specifically, it was shown to afford neuroprotection in models of cerebral ischemia (Spencer et al., 2013), Parkinson’s disease (Bayliss and Andrews, 2013), and *status epilepticus* (SE) (Xu et al., 2009; Lucchi et al., 2013). Interestingly, this last neuroprotective effect occurred at a dosage unable to alter the epileptic activity. However, ghrelin was also found to modulate seizures induced by several, different approaches (Casillas-Espinosa et al., 2012; Portelli et al., 2012a; Kovac and Walker, 2013; Clynen et al., 2014; Curia et al., 2014; Dobolyi et al., 2014). Independent studies suggested an anticonvulsant activity of ghrelin in experimental paradigms by which seizures were induced using pentylenetetrazole (Obay et al., 2007), penicillin (Aslan et al., 2009), kainic acid (Lee et al., 2010), and pilocarpine (Portelli et al., 2012b). According to Lee et al. (2010), the anticonvulsant effects of ghrelin could be related to the interaction of this peptide with its established receptor. However, the ghrelin receptor was also demonstrated to possess a proconvulsant activity in basal condition, whereas the observed anticonvulsant properties of ghrelin ligands were explained by the induction of ghrelin receptor internalization (Portelli et al., 2012b).

Other than ghrelin, ghrelin receptor agonists such as hexarelin (Biagini et al., 2011), capromorelin (Portelli et al., 2012b), and JMV-1843 (Coppens et al., 2016), the inverse agonists A778193 and [D-Arg^1^, D-Phe^5^, D-Trp^7,9^, Leu^11^] substance P (Portelli et al., 2012b), as well as the antagonist EP-80317 (Biagini et al., 2011), were all proven to be anticonvulsants. These data may suggest that all ghrelin receptor ligands could be able to desensitize neurons expressing the ghrelin receptor, so to block its proconvulsant activity (Portelli et al., 2012b). However, this hypothesis could not be supported in the case of the ghrelin receptor antagonist JMV-2959, which did not alter the induction of SE by pilocarpine (Biagini et al., 2011). Alternatively, it could be hypothesized that the variety of ghrelin-related peptides able to induce anticonvulsant effects may interact with different receptors that share a common anticonvulsant activity. Indeed, the existence of multiple ghrelin receptors has been hypothesized immediately following the discovery of ghrelin receptor (Davenport et al., 2005; Müller et al., 2015). In particular, it was evident that the protective effects exerted by ghrelin, des-acyl ghrelin and other related peptides in presence of an inflammatory reaction, especially in the cardiovascular system, were independent of the ghrelin receptor (Bujold et al., 2009, 2013; Bulgarelli et al., 2009).

EP-80317 (Haic-D-Mrp-D-Lys-Trp-D-Phe-Lys-NH_2_) is a hexapeptide with a primary structure similar to that of hexarelin, from which it differs for substitutions in the first and third amino acid residues causing the loss of the growth hormone (GH)-releasing properties possessed by the original peptide (Momany et al., 1981). EP-80317 is pharmacologically active on macrophages, and its activity on these cells was found to be dependent on activation of the peroxisome proliferator-activated receptor gamma (PPARγ). However, the ability to activate PPARγ is also common to ghrelin, des-acyl ghrelin and other ghrelin-related peptides, as all of them are able to interact with the CD36 scavenger receptor (Bujold et al., 2009, 2013). In the CNS, CD36 appears to be expressed prevalently although not exclusively in microglia (Glezer et al., 2009), whereas PPARγ is found in neurons of various cerebral regions, including those known to be involved in the generation and propagation of seizure activity, such as the hippocampus and the piriform cortex (Moreno et al., 2004; Warden et al., 2016). For this reason, we tried to elucidate whether PPARγ could be involved in the anticonvulsant effects of EP-80317. To this purpose, we designed experiments in which administration of EP-80317 was challenged by pretreatment with the PPARγ inhibitor GW9662 (Wong et al., 2015), in rodents exposed to seizure induction. Additionally, we characterized the expression of PPARγ in the hippocampus of mice treated with EP-80317.

## Materials and Methods

### Animals and Treatments

A total of thirty-seven adult male Sprague-Dawley rats (Harlan, San Pietro al Natisone, Italy), ranging 260–270 g of body weight, and 66 4-week-old male CD-1 mice (Charles River, Calco, Italy) were used in this study. All animals were housed in a specific pathogen-free facility under controlled environment with *ad libitum* access to water and food.

EP-80317 was obtained through conventional solid phase synthesis, dissolved in a physiologic solution and administered through intraperitoneal (i.p.) injection (330 μg/kg). GW9662 and dimethyl sulfoxide (DMSO) were purchased from Sigma–Aldrich Chemicals (St. Louis, MO, United States). GW9662 was dissolved in DMSO and diluted with saline and then administered (2 mg/kg, i.p.) (Rani et al., 2016).

All experiments were in compliance with the European Directive 2010/63/EU and carried out according to the national guidelines on animal experimental research of the Italian Ministry of Health (DM 126/2011 – B and DM 92/2013). The University of Modena and Reggio Emilia Ethics Committee approved the study protocol. All efforts were made to refine procedures to improve the welfare and to reduce the number of animals that were used.

### Experimental Design

For the pilocarpine model, we subdivided rats into four groups for behavior and video electrocorticographic (ECoG) recordings: (i) 10 control rats received saline (1 ml/kg, i.p.), (ii) 10 rats received EP-80317 alone, (iii) seven rats received GW9662 alone, and (iv) 10 rats received GW9662 10 min prior to EP-80317.

For the repeated 6-Hz corneal stimulation model, we considered a number of mice (*n* = 39) for behavior and video-ECoG recordings, and the others (*n* = 27) for immunohistochemical and immunofluorescence analysis. In particular, for behavior and video-ECoG recordings, we subdivided mice into the following groups: (i) nine control mice received saline (1 ml/kg, i.p.), (ii) 12 mice received EP-80317 alone (330 μg/kg, i.p.), (iii) nine mice received GW9662 alone (2 mg/kg, i.p.), and (iv) nine mice received GW9662 10 min prior to EP-80317, both at the already indicated doses. For immunohistochemical analyses, four control mice were used to determine basal levels of the investigated marker and were neither treated nor stimulated. Still, they were handled and exposed to the same procedure as the others. Twelve were saline-treated mice, while the other 11 mice received an i.p. EP-80317 injection. Moreover, these animals were used for qualitative immunofluorescence experiments but, in this case, we used only three mice per treatment group.

### Pilocarpine Protocol and Behavioral Analysis

Pilocarpine (Sigma–Aldrich, Milan, Italy) was injected i.p. (380 mg/kg) to induce SE. The pilocarpine injection was preceded by methylscopolamine (1 mg/kg, i.p.; Sigma–Aldrich) to prevent the peripheral effects of cholinergic stimulation (Curia et al., 2008). EP-80317, GW9662 alone, or saline were injected i.p. 20 min after methylscopolamine and 10 min before pilocarpine. In the group of rats treated with GW9662 and then with EP-80317, the first drug was administered 10 min prior to the second one. In all rats experiencing SE, diazepam (20 mg/kg, i.p.; Hospira Italia, Naples, Italy) was injected 10 min after the SE onset to guarantee survival. This procedure stops convulsive seizures, leaving non-convulsive SE unaltered for hours (Gualtieri et al., 2012).

Drug-induced responses in rats were observed directly and graded by blind to treatment expert raters. Seizures were graded according to a modification of the Racine’s scale (Racine, 1972). In particular, we considered: (i) non-convulsive seizures, ranked as stage 1 to stage 3; (ii) convulsive seizures, ranked as stage 4 to stage 5; (iii) SE (stage 6), considered as the stage in which rats either did not recover normal behavior (i.e., exploration, grooming, or motor reaction to stimuli) between one seizure and the other, or in which they displayed continuous shaking for more than 5 min (Lowenstein, 1999). Rats were sacrificed 72 h after SE to assess the absence of cerebral lesions caused by electrode implant, and the presence of injuries developed during the SE.

### Corneal Stimulation Protocol and Behavioral Analysis

Mice were stimulated once and allowed to recover for 72 h before being stimulated up to four sessions. Corneal stimulation was performed as previously described (Giordano et al., 2015, 2016). Briefly, a topical eye anesthetic (0.4% oxybuprocaine hydrochloride eye drops, Novesin, Novartis, Switzerland) was applied 10 min before stimulation. All mice received the injection of EP-80317, GW9662, or saline 10 min before each session of corneal stimulation. When GW9662 preceded EP-80317, it was administered 20 min before each session. Stimulation (fixed current intensity of 32 mA, pulse width of 0.2 ms, duration of 3 s, frequency of 6 Hz) was delivered via corneal electrodes connected to a stimulator (ECT Unit 5780; Ugo Basile, Comerio, Italy).

Seizure severity was ranked according to the following score: (i) stunned posture and eye blinking; (ii) head nodding, Straub tail and repetitive rhythmic movements (stereotypies) such as chewing; (iii) unilateral or alternating forelimb clonus; (iv) generalized tonic-clonic convulsions without loss of posture and rearings; (v) generalized tonic-clonic convulsions with loss of posture. Seizure scores were first recorded through direct observation, then reanalyzed on video recordings to quantify the duration of behavioral changes by an investigator unaware of the stimulation session. Recovery from seizures was defined as the reappearance of a normal exploratory behavior.

### Electrodes Implantation for Electrocorticographic (ECoG) Recordings

For electrode implantation, anesthesia was induced with volatile isoflurane (4% induction and 1–2% maintenance) in rats, whereas mice were anesthetized with ketamine (150 μg/g, i.p.) + xylazine (10 μg/g, i.p.). After deep anesthesia was reached (assessed by deep breath, loss of tail and eye reflexes), the skin was shaved, disinfected with povidone-iodine 10% (Betadine^®^ skin solution; Meda Pharma, Milano, Italy), cut and opened to expose the skull. Guiding holes were drilled and epidural electrodes (stainless steel Ø = 1 mm; PlasticsOne, Roanoke, VA, United States) were implanted in frontal (bregma 0 mm, 3.5 mm lateral from midline in rats and bregma 0 mm, 3 mm lateral from midline in mice) and occipital cortices (bregma -6.5 mm, 3.5 mm lateral from midline in rats and bregma -3.5 mm, 3 mm lateral from midline in mice) of both hemispheres. One electrode was implanted below the lambda on the midline in all animals and used as a reference. Electrodes were connected through steel wire to terminal gold pins (Bilaney Consultant GmbH, Düsseldorf, Germany) inserted in a plastic pedestal (PlasticsOne) cemented on heads. At the end of the surgery, gel containing 2.5 g lidocaine chloride, 0.5 g neomycin sulfate and 0.025 g fluocinolone acetonide (Neuflan^®^ gel; Molteni Farmaceutici, Scandicci, FI, Italy) was applied to reduce acute pain and risk of infection. All animals were monitored until complete recovery from anesthesia, and were housed in single cages without grids or environmental enrichments to reduce risk of headset loss.

### Video ECoG Recordings

For brain activity recording, animals were placed in cages with paper filter cover that allowed cable connection between headset and preamplifiers. Electrical brain activity was digitally filtered (0.3 Hz high-pass, 500 Hz low-pass), acquired at 1 kHz per channel, and stored on a personal computer after the mathematical subtraction of traces of recording electrodes from trace of reference electrode, using a PowerLab8/30 amplifier connected to 4 BioAmp preamplifiers (ADInstruments; Dunedin, Otago, New Zealand). Videos were digitally captured through a camera connected to the computer and synchronized to the ECoG traces through LabChart 7 PRO internal trigger. Four days after electrode implantation, all animals were connected to the recording system and received the sequence of treatments described above. To facilitate handling and pharmacological manipulations, recordings were stopped, animals were temporarily disconnected while being injected and reconnected soon after. In particular, rats were recorded for 20 min after methylscopolamine administration, 10 min after EP-80317, GW9662 or saline injection, 10 min after SE onset and at least 2 h after diazepam administration. Rats that did not experience SE were recorded for at least 90 min after pilocarpine injection, until signs of normal behavior (exploring, grooming) reappeared. Mice were recorded after EP-80317, GW9662 or saline injection and 6-Hz corneal stimulation, until signs of normal behavior reappeared.

### ECoG Analysis

Electrocorticographic traces were digitally filtered offline (band-pass: high 50 Hz, low 1 Hz) and manually analyzed using LabChart 7 PRO software (AD Instruments) by expert raters.

In rats, we measured the duration of each electrographic seizure, characterized as epileptiform ECoG patterns with trains of 150–250 ms long spikes with amplitudes at least twice as the previous 2 s baseline. Seizures occurring within 5 s of each other were defined as one epileptic event.

In mice, we quantified the ECoG signal of each seizure performing a power spectral analysis, that described the distribution of signal power over frequency (Dressler, 2004). In particular, the power spectrum was obtained by fast Fourier transformation of the ECoG waveforms (LabChart 7 PRO, ADInstruments) and used to obtain the mean power spectra for the experimental groups. The maximum value of power spectra (peak value) was considered for each mouse. Peak values of power, recorded from frontal and occipital electrodes, were evaluated to investigate whether modifications of ictal activity had occurred from the first to the fourth stimulation.

### Immunohistochemistry

Control mice and mice receiving up to one or three sessions of 6-Hz corneal stimulation and treated with saline or EP-80317 were used for tissue analysis. Mice deeply anesthetized with isoflurane were transcardially perfused with phosphate buffered saline (PBS, pH 7.4) followed by Zamboni’s fixative (pH 6.9), 14–17 h after the seizure. Brains were post-fixed at 4°C in the same fixative for 24 h, cryoprotected in 15 and 30% sucrose solutions (Vinet et al., 2016) and stored at -80°C until used. Horizontal sections of 50 μm were cut using a freezing sliding microtome (Leica SM2000 R; Leica, Nussloch, Germany). For immunostaining, sections were washed in Tris-buffered saline (TBS) and incubated in 3% H_2_O_2_ in TBS (30 min) to quench endogenous peroxidase activity. Following another washing step, sections were blocked 1 h in TBS containing 2% bovine serum albumin, 0.3% Triton X-100 (Tx) and 5% normal goat serum. Sections were then placed at 4°C with a polyclonal rabbit anti-PPARγ (Ab209350; Abcam, Cambridge, United Kingdom, dilution 1:2000, 48 h). After washing, sections were incubated for 1 h with a biotinylated anti-rabbit secondary antibody (Vector Laboratories, Burlingame, CA, United States; 1:200), and later with the avidin-biotin-peroxidase complex (Elite ABC Kit; Vector Laboratories, Burlingame, CA, United States). The immunostaining was performed in 0.05% 3,3-diaminobenzidine tetrahydrochloride for 5 min (DAB, Sigma–Aldrich, Milan, Italy) and developed by adding 0.03% H_2_O_2_. Finally, sections were washed again in TBS, mounted on gelatin-coated slides and cover slipped with Eukitt (Eukitt^®^, O. Kindler GmbH & Co., Freiburg, Germany).

### Image Analysis

Immunostained sections related to bregma from -8.04 mm to -5.04 mm for hippocampal CA1 and CA3 regions, and hilus of the dentate gyrus (DH) were analyzed using an Axioskop microscope (Carl Zeiss Vision GmbH, Munchen, Germany) equipped with a 10X objective. Images were digitally captured by a Sony CCD-IRIS B–W video camera, along the ventrodorsal direction of the brain (approximately 6–7 serial horizontal sections separated by 0.5 mm). A mouse brain atlas-C57BL/6J horizontal was used to assess brain sections. The image analysis was carried out using the KS300 software (Carl Zeiss Vision GmbH), as previously described (Biagini et al., 2005; Curia et al., 2013; Giordano et al., 2015, 2016). PPARγ immunoreactivity was measured as field area values, corresponding to the addition of areas of the specific profiles obtained after discrimination from background staining. Background values in each section were obtained from areas devoid of specific immunostaining, such as the angular bundle. All measurements were taken bilaterally and the final values represent the left–right average.

### Double Immunofluorescence

We performed a PPARγ/67-kDa glutamate decarboxylase (GAD67) qualitative double immunofluorescence to assess PPARγ expression in interneurons. PPARγ expression was further evaluated in different interneuron subsets by PPARγ/parvalbumin (PV), PPARγ/somatostatin-28 (SOM) or PPARγ/vasoactive intestinal peptide (VIP) qualitative double immunofluorescences. For PPARγ/GAD67 immunofluorescence, sections were washed in TBS at room temperature and unmasking in sodium citrate at 98°C and permeabilized for 1 h in TBS/0.02% Triton X-100 containing 5% normal goat serum. Concerning PPARγ/PV, PPARγ/SOM and PPARγ/VIP immunofluorescences, sections were washed in TBS at room temperature and permeabilized for 1 h in TBS/0.1% Triton X-100 containing 5% normal goat serum. Then, sections were incubated for 48 h with primary antibodies: rabbit anti-PPARγ (Ab209350; Abcam, Cambridge, United Kingdom, dilution 1:50), mouse anti-GAD67 (MAB5406; Chemicon International, Billerica, MA, United States, dilution 1:200), mouse anti-PPARγ (E-8, sc-7273; Santa Cruz Biotechnology, Santa Cruz, CA, United States, dilution 1/50), rabbit anti-PV (Ab11427; Abcam, Cambridge, MA, United States, dilution 1:2000), rabbit anti-SOM (20089; Immunostar, Hudson, WI, United States, dilution 1/1000) and rabbit anti-VIP (20077; Immunostar, Hudson, WI, United States, dilution 1/500). Following a washing step in TBS, sections were incubated for 3 h at room temperature with secondary antibodies Alexa Fluor 488-labeled goat anti-mouse antibody (A-11001; Invitrogen, Carlsbad, CA, United States, dilution 1:500) and Alexa Fluor 594-labeled goat anti-rabbit (A-11012; Invitrogen, Carlsbad, CA, United States, dilution 1:500). After rinsing sections in TBS, brain sections were placed on gelatinized glass slides, dried, and mounted with Mowiol after incubation with DAPI. Images were acquired using a Leica TCS SP2 (Leica Laser Technik, Heidelberg, Germany) confocal microscope. All images were taken using a 40X magnification.

### Statistics

Chi-Square test (χ^2^) was used to compare the percentage (%) of rats experiencing SE in the various treatment groups. Pairwise comparisons with Fisher’s exact test (using α correction) were used to establish differences among groups. Additionally, the time interval (min) required to develop seizures and SE in the various groups was analyzed by Kruskal–Wallis test. Dunn’s test was used for multiple comparisons. One-way analysis of variance (ANOVA) was used to compare electrographic seizure duration (sec) in each treatment groups.

The χ^2^ test was also used to compare mice experiencing seizures with loss of posture in the various treatment groups. Again, pairwise comparisons with Fisher’s exact test (using α correction) were used to establish differences among groups. Electrocorticographic recordings, analyzed as peak values of power spectra, were compared using two-way ANOVA, considering treatments as the between-factor and sessions as the within-factor. The Holm-Šídák test was used for multiple comparisons. All statistical analyses were performed using Sigmaplot 11 (Systat Software, San Jose, CA, United States). Unless otherwise indicated, results are shown as mean ± standard error of the mean (SEM); *p*-values lower than 0.05 were considered as statistically significant.

## Results

### EP-80317 Reduced the Percentage of Animals Developing SE after Pilocarpine Injection

Response to pilocarpine was assessed by expert raters who directly annotated the motor responses observed during seizure development according to the modified Racine’s scale (Racine, 1972; Lucchi et al., 2013), and by offline analysis of video-EcoG recordings. Four (*n* = 1 saline group, *n* = 2 EP-80317 group, and *n* = 1 GW9662 group) animals died during continuous tonic-clonic seizures and were excluded from the analysis. Consistently with previous findings (Biagini et al., 2011), pilocarpine induced SE in 100% of saline-treated rats. Instead, only 50% of rats treated with EP-80317 developed SE (*p* < 0.05 vs. saline-treated rats, Fisher’s exact test). Additionally, we also found that the percentages of rats developing SE after pilocarpine administration in groups treated with GW9662 or GW9662+EP-80317 (respectively, 83% and 80%) were not significantly different from that observed in saline-treated rats (**Table 1**).

**Table 1:** Percentage (%) of rats developing *status epilepticus* (SE) in the various treatment groups.

Treatments	Percentage (%) of rats developing SE
Saline (*n* = 9)	100%
EP-80317 (EP, *n* = 8)	50%^∗^
GW9662 (GW, *n* = 6)	83%
GW+EP (*n* = 10)	80%


*^∗^*p* < 0.05, EP-80317 vs. saline; Fisher’s exact test.*

### GW9662 Accelerated the Development of Convulsive Seizure and SE in the Pilocarpine Model

We calculated the time interval required to develop the first stage 1-3 and stage 4-5 seizure after pilocarpine administration, as median and interquartile range (IRQ) values. Moreover, we calculated the time interval required to develop SE (**Table 2**). No significant differences were found when comparing the latency periods of the first stage 1-3 seizure in the various treatment groups. Instead, the latency periods for developing the first stage 4-5 seizure were significantly different in GW9662-treated or GW9662+EP-80317-treated rats compared to, respectively, saline-treated rats (*p* < 0.05 for both groups, Dunn’s test) and the EP-80317 group of treatment (*p* < 0.05). In addition, the latency periods for developing SE after the first stage 4-5 seizure were significantly shorter in GW9662-treated and GW9662+EP-80317-treated rats compared to saline-treated rats (*p* < 0.05).

**Table 2:** Median and interquartile range values of latency periods for developing seizure and *status epilepticus* (SE) in the various treatment groups.

Latency (min)	Saline	EP-80317 (EP)	GW9662 (GW)	GW+EP
From pilocarpine injection to first stage 1-3 seizure	2.00 (1.75–3.50)	4.00 (3.00–11.75)	3.00 (2.00–4.00)	3.00 (3.00–4.00)
From pilocarpine injection to first stage 4-5 seizure	9.00 (6.75–10.25)	18.00 (8.00–21.25)	3.00 (2.00–4.00)^∗§^	3.00 (3.00–4.00)^∗§^
From pilocarpine injection to SE	17.00 (16.00–18.50)	13.00 (10.75–25.00)	14.00 (11.00–14.50)	16.50 (14.50–21.75)
From first stage 4-5 seizure to SE	8.00 (8.00–10.50)	5.50 (2.75–6.75)	3.00 (2.50–4.50)^#^	3.50 (3.00–6.50)^#^


*^∗^*p* < 0.05, GW9662 vs. saline and GW9662+EP-80317 vs. saline; Dunn’s test. ^§^*p* < 0.05, GW9662 vs. EP-80317 and GW9662+EP-80317 vs. EP-80317. ^#^*p* < 0.05, GW9662 vs. saline and GW9662+EP-80317.*

### Drug Treatments Did Not Affect Seizure Duration in Pilocarpine-Treated Rats

**FIGURE 1:**
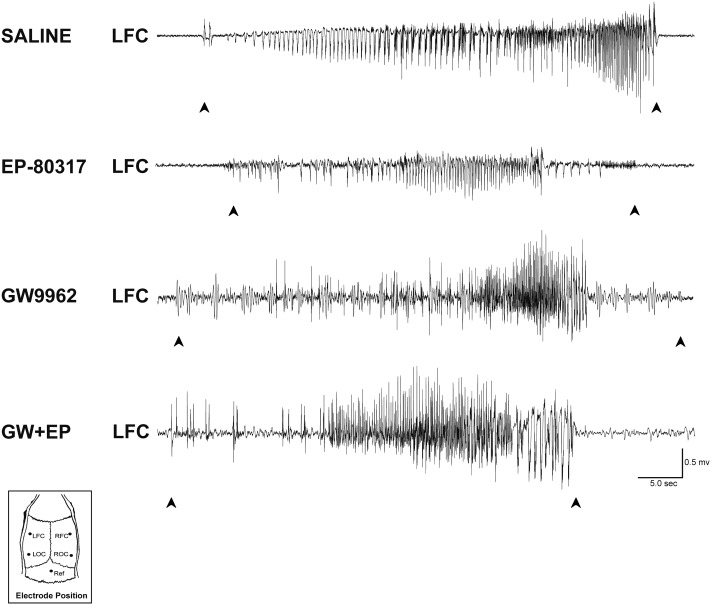
ECoG analysis of seizure duration in the pilocarpine model. Representative epileptiform activity recorded from each treatment group is shown. In saline-treated and GW9662-treated rats, the seizure duration was similar to those of EP-80317 and GW9662+EP-80317 (GW+EP) groups. LFC, left frontal cortex; LOC, left occipital cortex; Ref, reference electrode; RFC, right frontal cortex; ROC, right occipital cortex.

We measured the average seizure duration in traces obtained from ECoG recordings (**Figure 1**) performed in the various groups of treatment. No differences were found for animals treated, respectively, with saline (65.32 ± 8.75 s) or GW9662 (67.15 ± 10.95 s). Although the EP-80317 group presented a lower seizure duration (45.75 ± 9.24 s), this difference did not reach a statistically significant level. Mean seizure duration was also lower (49.91 ± 12.64 s) in GW9662+EP-80317 but, again, not enough to be statistically relevant.

### EP-80317 Transiently Reduced the Seizure Severity and Duration in the Repeated 6-Hz Corneal Stimulation Model

**FIGURE 2:**
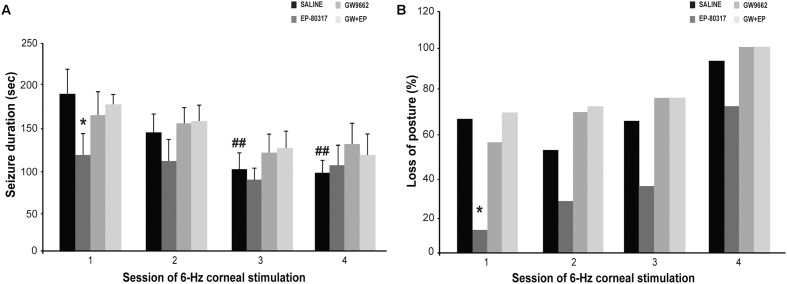
Behavioral changes observed during repeated 6-Hz corneal stimulation. Seizure duration and severity are respectively illustrated in **(A,B)** for the various treatment groups. **(A)** The PPARγ activator EP-80317 significantly reduced the duration of seizures induced by the first 6-Hz corneal stimulation (^∗^*p* < 0.05, EP-80317 vs. saline in the first session of stimulation; Holm-Šídák test). Administering GW9662, a PPARγ blocker, counteracted this effect (GW+EP group). Note also that seizure duration significantly decreased in control mice at the third and fourth sessions (^##^*p* < 0.01, sessions three or four vs. session one in saline-treated mice; Holm-Šídák test). **(B)** The seizure severity, evaluated as the percentage of animals displaying postural loss during convulsions, was also significantly affected by EP-80317 (^∗^*p* < 0.05, EP-80317 vs. saline in the first session of stimulation; Fisher’s exact test).

Experimenters first visually monitored seizures induced through 6-Hz corneal stimulation and their pharmacological modulations. The first seizure duration was significantly shorter in mice treated with EP-80317 compared to control mice (*p* < 0.05, Holm-Šídák test), thus confirming the anticonvulsant properties of this molecule (Biagini et al., 2011; Giordano et al., 2016). No differences were noticed by comparing controls to mice treated with GW9662 alone or prior to EP-80317 (**Figure 2A**). Similarly seizure severity, defined as the percentage of mice displaying loss of posture during seizures, was also less pronounced in the presence of EP-80317 (*p* < 0.05, EP-80317 vs. saline in the first session of stimulation; Fisher’s exact test). No differences were observed by comparing the other groups with saline-treated mice (**Figure 2B**).

As expected (Giordano et al., 2015), seizures significantly shortened by repeating 6-Hz corneal stimulation (*p* < 0.01, sessions three and four vs. session one in saline-treated mice; Holm-Šídák test). Consequently, no differences between mice treated with saline and EP-80317 were observed from the second session onward (**Figure 2A**). Moreover, the severity of seizures progressed in all groups, including mice treated with EP-80317 (**Figure 2B**), which confirmed the appearance of resistance to the effects of EP-80317 (Giordano et al., 2016). Consistently, in the fourth session of 6-Hz corneal stimulation no differences were present within the various treatment groups.

### EP-80317 Prevented the Increase in Power Spectra of Seizures Induced by Repeated 6-Hz Corneal Stimulation

**FIGURE 3:**
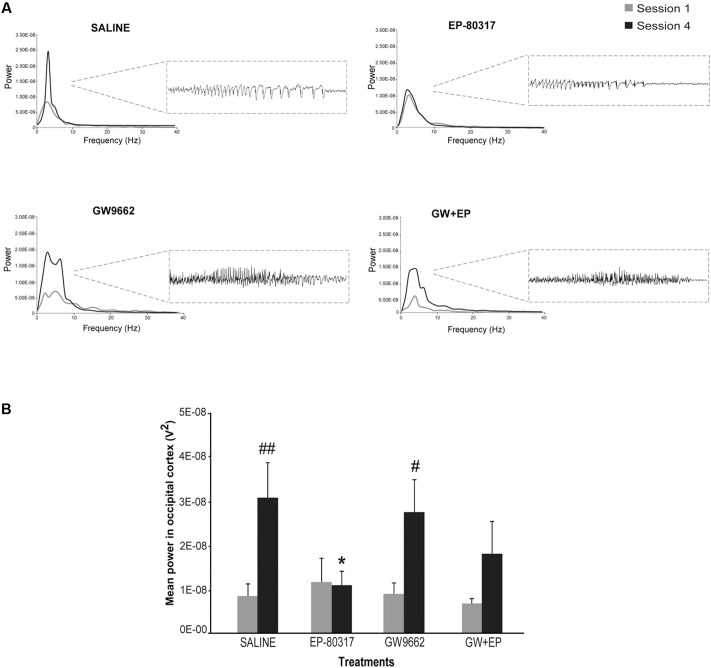
Electrographic changes observed during repeated 6-Hz corneal stimulation. **(A)** Representative power spectra obtained at session one and four are illustrated for each treatment group. Note that powers of ictal activity did not significantly change going from first to fourth stimulation in mice treated with EP-80317, whereas clear alterations were present in all other groups. The inset on the right shows electrographic traces corresponding to power spectra of traces recorded by the occipital electrodes, after the fourth seizure induction. The gray color is for the first session; the black color is for the fourth session. **(B)** Mean power spectra of ictal events obtained through one or four separated 6-Hz corneal stimulation sessions. Histograms show that the power peak of ictal events significantly increased at the fourth session in saline-treated mice (^##^*p* < 0.01, session 4 vs. session 1; Holm-Šídák test) and in mice treated with GW9662 (^#^*p* < 0.05, session 4 vs. session 1), but not in mice treated with EP-80317 alone or in combination with GW9662 (GW+EP). Note also that the mean power peak of animals treated with EP-80317 was significantly lower than that of saline-treated mice, at the fourth stimulation (^∗^*p* < 0.05, EP-80317 vs. saline in the fourth session of stimulation).

Seizures induced by 6-Hz corneal stimulation and their pharmacological modulations were further monitored through video ECoG recordings. In particular, we compared power spectra of seizures recorded from frontal (not shown) and occipital electrodes (**Figure 3A**). As previously reported (Giordano et al., 2015), power peaks did not change for ictal events recorded through frontal cortex electrodes, in all groups, whereas in the occipital recording we noticed the presence of an epileptogenic process. In particular, we found a significant increase in power peaks recorded from occipital electrodes in the fourth session, both in saline-treated and GW9662-treated mice (*p* < 0.01 for saline-treated mice; *p* < 0.05 for GW9662-treated mice, session four vs. session one; Holm-Šídák test; **Figure 3B**). Conversely, power peaks in mice treated with EP-80317 did not change from the first to the fourth session of 6-Hz corneal stimulation, suggesting an antiepileptogenic effect. This effect was not completely prevented by administering GW9662 prior to EP-80317, although a trend was present (*p* = 0.09). Interestingly, significantly lower values were found in the fourth session of mice treated with EP-80317 compared to saline-treated mice (*p* < 0.05; **Figure 3B**).

### EP-80317 Significantly Increased PPARγ Immunoreactivity in the Hippocampus of Mice Exposed to the First 6-Hz Corneal Stimulation

**FIGURE 4:**
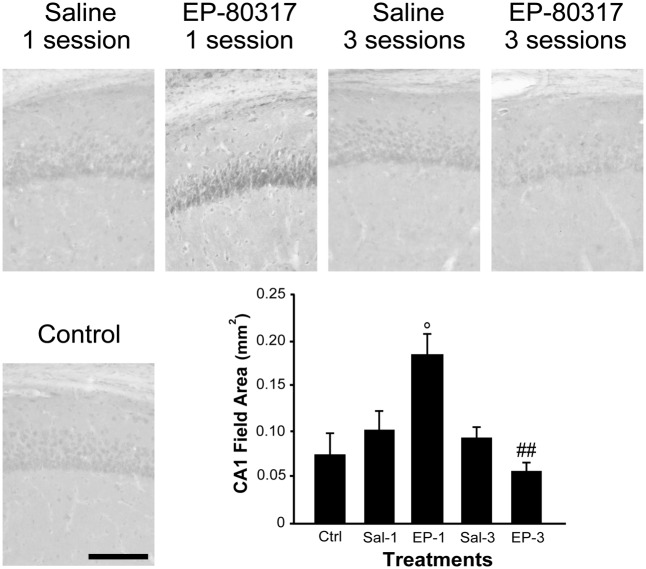
PPARγ immunoreactivity in the hippocampal CA1 region of mice treated with saline or EP-80317 and exposed to different sessions of 6-Hz corneal stimulation. PPARγ immunoreactivity is illustrated in a representative unstimulated control (Ctrl) mouse and in mice treated with saline (Sal) or EP-80317 (EP) and respectively exposed to one (Sal-1, EP-1) or three (Sal-3, EP-3) different sessions of 6-Hz corneal stimulation. Note that PPARγ levels were significantly increased after the first session in EP-80317 mice only (°*p* < 0.05, EP-1 vs. Ctrl; Holm-Šídák test). Note also that PPARγ levels were significantly reduced in the third session of EP-80317 mice (^##^*p* < 0.01, EP-3 vs. EP-1). Scale bar: 100 μm.

**FIGURE 5:**
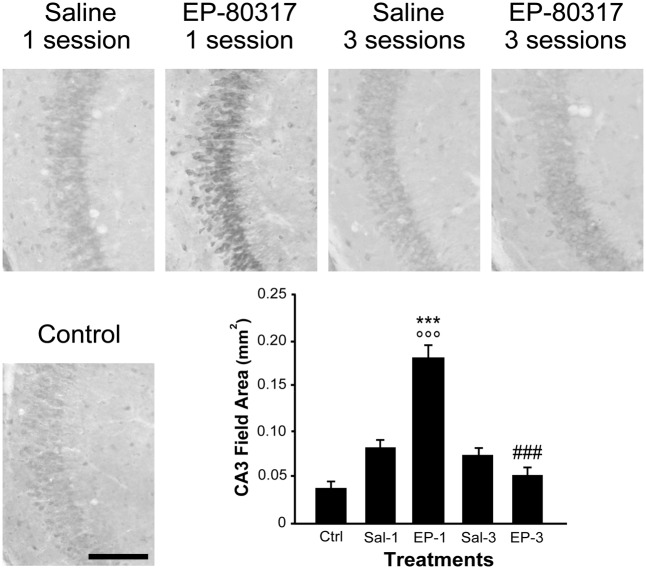
PPARγ immunoreactivity in the hippocampal CA3 region of mice treated with saline or EP-80317 and exposed to different sessions of 6-Hz corneal stimulation. PPARγ immunoreactivity is illustrated in a representative unstimulated control (Ctrl) mouse and in mice treated with saline (Sal) or EP-80317 (EP) and respectively exposed to one (Sal-1, EP-1) or three (Sal-3, EP-3) different sessions of 6-Hz corneal stimulation. Note that PPARγ levels were significantly increased after the first session in EP-80317 mice only (^∘∘∘^*p* < 0.001, EP-1 vs. Ctrl; Holm-Šídák test). In EP-80317 mice, PPARγ levels were also significantly different from saline-treated mice (^∗∗∗^*p* < 0.001, EP-1 vs. Sal-1 in CA3). Note also that PPARγ levels were significantly reduced in the third session of EP-80317 mice (^###^*p* < 0.001, EP-3 vs. EP-1). Scale bar: 100 μm.

**FIGURE 6:**
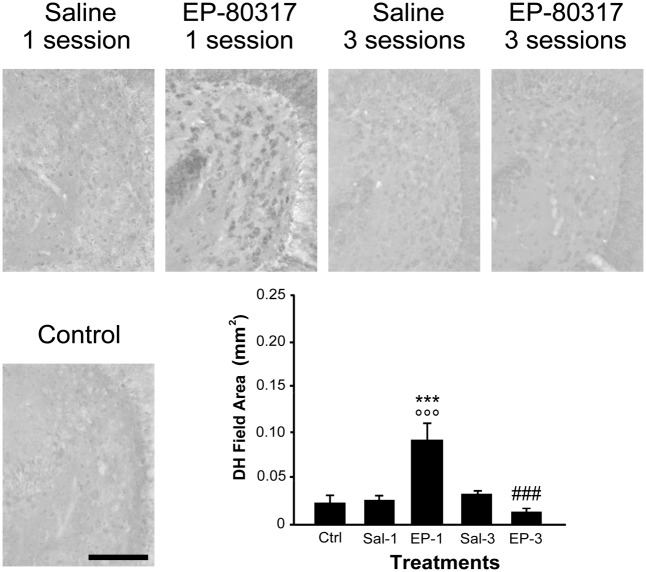
PPARγ immunoreactivity in the hilus of dentate gyrus (DH) of mice treated with saline or EP-80317 and exposed to different sessions of 6-Hz corneal stimulation. PPARγ immunoreactivity is illustrated in a representative unstimulated control (Ctrl) mouse and in mice treated with saline (Sal) or EP-80317 (EP) and respectively exposed to one (Sal-1, EP-1) or three (Sal-3, EP-3) different sessions of 6-Hz corneal stimulation. Note that PPARγ levels were significantly increased after the first session in EP-80317 mice only (^∘∘∘^*p* < 0.001, EP-1 vs. Ctrl; Holm-Šídák test). PPARγ levels were also significantly different from saline-treated mice (^∗∗∗^*p* < 0.001, EP-1 vs. Sal-1). Note also that PPARγ levels were significantly reduced in the third session of EP-80317 mice (^###^*p* < 0.001, EP-3 vs. EP-1). Scale bar: 100 μm.

We evaluated the effects of repeated exposure to 6-Hz corneal stimulation on PPARγ immunoreactivity in the *stratum pyramidalis* of CA1 (**Figure 4**), in both *stratum pyramidalis* and *stratum radiatum/lacunosum-moleculare* of CA3 (**Figure 5**), and finally in the DH (**Figure 6**). First, we quantified the changes occurring after the first and third session of seizure induction in pyramidal cells of the hippocampus and in mossy cells of the DH. PPARγ levels were scantily detectable in all sampled regions of unstimulated control mice. PPARγ immunoreactivity was not significantly changed in regions of interest of saline-treated mice. Notably, in animals treated with EP-80317 we observed a prominent increase in PPARγ levels after the first 6-Hz corneal stimulation, reaching a statistically significant level in all the considered regions (*p* < 0.05 vs. controls, in CA1; *p* < 0.001 vs. controls and saline-treated mice, in CA3 and DH; Holm-Šídák test; **Figures 4**–**6**). After the third seizure induction, PPARγ immunoreactivity returned to basal levels in all hippocampal regions.

To evaluate the response in interneurons, we analyzed the changes occurring in the *stratum radiatum/lacunosum-moleculare* of the CA3 (**Table 3**). Again, PPARγ levels were barely detectable in unstimulated control mice and saline-treated mice, but a significant increase in PPARγ levels was found in mice treated with EP-80317 after the first 6-Hz corneal stimulation (*p* < 0.01 vs. controls; *p* < 0.05 vs. saline-treated mice, Holm-Šídák test; **Table 3**). Consistently, PPARγ levels returned to basal levels after the third seizure induction.

**Table 3:** EP-80317 (EP) significantly increased PPARγ immunoreactivity in interneurons within the CA3 *stratum radiatum/lacunosum-molecolare* of mice exposed to the first 6-Hz corneal stimulation (EP-1), but not in mice exposed to the third stimulation (EP-3), compared to saline-treated mice (Sal) and controls (Ctrl).

Treatment groups	Field area (mm^2^) mean values	SEM
Ctrl (*n* = 4)	0.016	0.0016
Sal-1 (*n* = 5)	0.020	0.0021
EP-1 (*n* = 5)	0.035^∘∘∗^	0.0055
Sal-3 (*n* = 7)	0.018	0.0009
EP-3 (*n* = 6)	0.010^###^	0.0018


*^∗^*p* < 0.05, EP-1 vs. Sal-1; Holm-Šídák test; ^∘∘^*p* < 0.01, EP-1 vs. Ctrl; ^###^*p* < 0.001, EP-3 vs. EP-1.*

### PPARγ Is Expressed in Different Subsets of Interneurons

**FIGURE 7:**
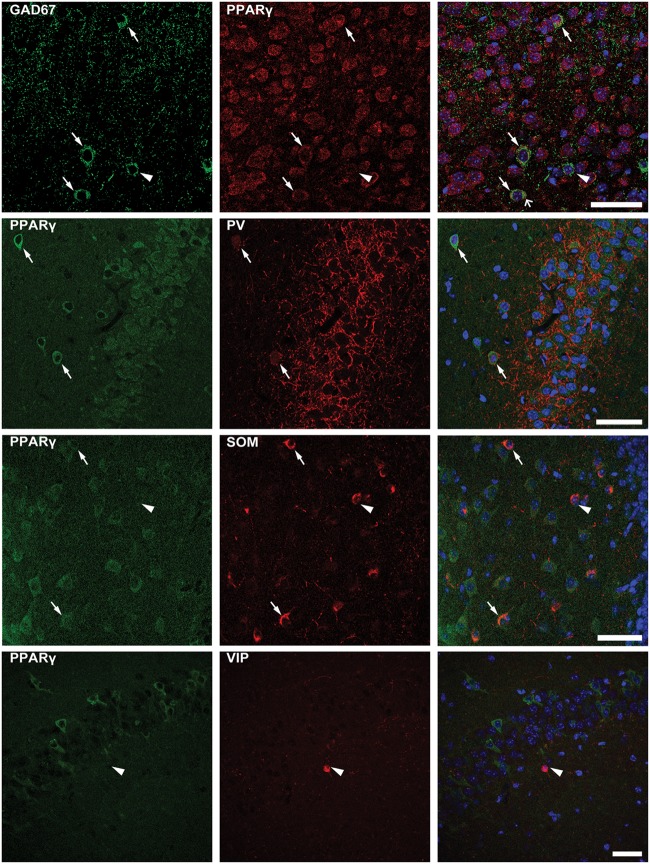
Double immunofluorescence of interneurons expressing PPARγ. Photomicrographs illustrating co-labeling with glutamate decarboxylase 67 (GAD67, green) and PPARγ (red) in CA1 of a representative mouse are shown in top panels. Double immunofluorescence revealed the co-expression of GAD67 and PPARγ in some (arrows), but not all interneurons (arrowhead point to a PPARγ negative interneuron). Scale bar: 50 μm. Photomicrographs illustrating co-labeling of PPARγ (green) and parvalbumin (PV, red), somatostatin (SOM, red), or vasoactive intestinal peptide (VIP, red) in the hippocampus of mice exposed to repeated 6-Hz corneal stimulation are sequentially shown from the second row to bottom. Note that double immunofluorescence revealed the co-labeling of PPARγ/PV in CA3, PPARγ/SOM in the hilus of dentate gyrus (DH) (arrows), but not PPARγ/VIP in CA1 (arrowhead). Scale bar: 50 μm.

To confirm that PPARγ was expressed in interneurons, we performed a double immunofluorescence experiment with PPARγ and GAD67 antibodies. We found that not all interneurons coexpressed GAD67 and PPARγ immunoreactivity (**Figure 7**, top panels). Thus, to assess the subset of interneurons expressing PPARγ immunoreactivity, we evaluated PPARγ/PV, PPARγ/SOM and PPARγ/VIP colabeling. PPARγ-positive cells were frequently observed within PV interneurons (**Figure 7**, in which arrows indicate colabeling), and less frequently identified within SOM interneurons. In contrast, PPARγ never colocalized with VIP in interneurons (**Figure 7**, in which arrowheads indicate lack of colabeling).

## Discussion

In the present investigation we confirmed that EP-80317 displays anticonvulsant effects in the pilocarpine model and in the repeated 6-Hz corneal stimulation model. As major findings of our investigation, we also demonstrated that GW9662, an inhibitor of PPARγ activity, is able to counteract the anticonvulsant effects of EP-80317 in both the animal models, with the notable exception of the antiepileptogenic properties disclosed by analyzing the ECoG power spectrum in mice. We also observed a proconvulsant activity of GW9662 when analyzing the latency period of stage 4-5 seizures, and the latency period of SE onset after the first stage 4-5 seizure in the pilocarpine model. Interestingly, PPARγ immunoreactivity was transiently increased in pyramidal cells and interneurons belonging to PV and SOM subclasses, in coincidence with the anticonvulsant effects of EP-80317 in 6-Hz corneally stimulated mice.

We previously established that the ghrelin receptor antagonist EP-80317 is an anticonvulsant in the pilocarpine model (Biagini et al., 2011), as well as in the repeated 6-Hz corneal stimulation model (Giordano et al., 2016). We also hypothesized that its anticonvulsant activity could not be explained by an involvement of the ghrelin receptor, as we did not observe any anticonvulsant activity of either the ghrelin receptor antagonist JMV-2959 (Biagini et al., 2011), nor of ghrelin, and of the ghrelin receptor agonist JMV-1843 (Biagini et al., 2011; Lucchi et al., 2013), all administered i.p. 10 min before injecting pilocarpine. At odds with our data, however, other investigators consistently showed that ghrelin produces some anticonvulsant effects in different seizure models (Portelli et al., 2012a,b). Furthermore, JMV-1843 also was found to be an anticonvulsant when used at high doses (Coppens et al., 2016). So, technical reasons, such as the more direct intracerebroventricular route of ghrelin administration in some cases (Portelli et al., 2012b), the earlier timing of ghrelin injection in others (Obay et al., 2007; Lee et al., 2010; Portelli et al., 2012b), and the different doses of the tested ghrelin receptor agonist (Coppens et al., 2016) may help in understanding why ghrelin receptor agonists in our hands did not display anticonvulsant properties (Biagini et al., 2011; Lucchi et al., 2013). Alternatively, it could be proposed that different receptors are involved in the anticonvulsant effects of ghrelin and its related peptides.

EP-80317 was found to activate PPARγ, as well as its cascade in apolipoprotein E-deficient mice (Bujold et al., 2009, 2013; Bulgarelli et al., 2009). Although PPARγ was mainly localized in the liver and in adipocytes, in which it was characterized for its role in metabolism regulation (Demers et al., 2008; Li et al., 2014; Gorga et al., 2017), it was also found that PPARγ agonists such as rosiglitazone or pioglitazone display antiseizure effects (Adabi Mohazab et al., 2012; Hong et al., 2013). It has also been proposed that PPARγ plays a key role in the antiseizure effects of cannabinoid agonists (Payandemehr et al., 2015), as well as in the anticonvulsant effects of the ketogenic diet (Jeong et al., 2011; Simeone et al., 2017). In agreement with these suggestions, our data support the involvement of PPARγ in the anticonvulsant effects of EP-80317. Consistently, by inhibiting PPARγ with the antagonist GW9662, the effects of rosiglitazone on seizure-like activity induced by Mg^2+^-free medium in hippocampal slices were partially prevented (Wong et al., 2015). In line with these *in vitro* studies, the effects of EP-80317 were counteracted by GW9662 in our *in vivo* experiments and, additionally, we have identified a proconvulsant effect of GW9662 in the pilocarpine model. Indeed, as GW9662 produced a decrease in the latency period of tonic-clonic seizures and accelerated the onset of SE, a question arises on whether the GW9662 and EP-80317 combination resulted in the simple summation of their independent pro/anticonvulsant effects or not, at least in the pilocarpine model.

The suggested ability to modulate seizures of PPARγ could be explained by the fact that this nuclear receptor is also expressed in peripheral immune cells, microglia, and neurons (Moreno et al., 2004; Bujold et al., 2009; Warden et al., 2016). Especially by acting on immune cells, PPARγ exerts a negative modulation of macrophages and microglia reactivity (Moreno et al., 2004; Bujold et al., 2009; Warden et al., 2016), resulting in an anti-inflammatory action in the nervous tissue (Jeong et al., 2011). This effect may be important as several lines of evidence suggest that leukocytes and microglia are critical in establishing the onset of SE in the pilocarpine model (Fabene et al., 2008; Vezzani et al., 2015; Vinet et al., 2016). In particular, treatment with antibodies against leukocyte α_4_ integrin, which is involved in inflammation, successfully prevented the onset of SE in pilocarpine-treated mice (Fabene et al., 2008). Additional evidences indicate that inflammation is required to establish the SE in pilocarpine-treated rodents (Marchi et al., 2009; Vezzani et al., 2015). Overall, these findings suggest that EP-80317 activation of the PPARγ pathway in immune cells (Bujold et al., 2009, 2013; Bulgarelli et al., 2009) may have contributed to prevent the SE in pilocarpine-treated rats. However, we cannot rule out the possibility that other ghrelin analogs could share the same mechanism found for EP-80317. Indeed, further experiments are required to establish if PPARγ may mediate the anticonvulsant effects of other ghrelin receptor agonists or antagonists.

The effects of EP-80317 on PPARγ could be dependent on a direct modulation of the neuronal activity. As a matter of fact, EP-80317 markedly increased PPARγ immunoreactivity in hippocampal pyramidal neurons and also in interneurons of 6-Hz corneally stimulated mice. It is interesting to notice that we have identified PPARγ in interneurons and described its expression in different interneuron subpopulations for the first time. Recently, the role of interneurons in seizure initiation and propagation has become more controversial (de Curtis and Avoli, 2016). In particular, the different interneuron subclasses were suggested to play specific roles in modulating seizures (Khoshkhoo et al., 2017). Here, we found that PV and SOM but not VIP interneurons expressed PPARγ and, thus, were probably involved in the anticonvulsant effects of EP-80317. Interestingly, optogenetic inhibition of VIP interneurons affected both the seizure threshold and duration, whereas PV and SOM interneuron inhibition apparently reduced only the seizure duration (Khoshkhoo et al., 2017). According to these data, it is possible that the effects on seizure duration observed with EP-80317 in 6-Hz stimulated mice could be partly mediated by modulation of PV and SOM interneurons. It could also be of interest to evaluate other PPARγ modulators, such as rosiglitazone or pioglitazone, in the pilocarpine model and, especially, in the repeated 6-Hz corneal stimulation model to examine their effects in exactly the same experimental conditions in which we characterized EP-80317.

Although all the EP-80317 anticonvulsant effects were counteracted by GW9662, a notable exception was found by analyzing the ECoG power spectra of 6-Hz corneally stimulated mice. In particular, we observed that the power of ECoG recordings increased significantly in saline-treated and GW9662-treated mice, but not in mice receiving EP-80317 or GW9662+EP-80317. These findings suggest that the already proposed antiepileptogenic activity of EP-80317 (Giordano et al., 2016) may be independent of PPARγ activation. This hypothesis is in line with the time course of PPARγ induction in the hippocampus, which has been characterized by a transient increase at the first session of seizure induction returning to basal levels at the third session. So, PPARγ was presumably restored at basal levels when the effect of EP-80317 on the power of ECoG recordings was still present. Although we could not establish if this last finding was related to antagonism at the ghrelin receptor, it was certainly worth to further investigate this pharmacological activity of EP-80317 in view of the well-recognized need of antiepileptogenic drugs to prevent the appearance of epilepsy in subjects exposed to this risk, as in the case of patients which develop SE and, for this reason (Santamarina et al., 2015), may become affected by epilepsy.

## Author Contributions

GB is responsible for the experimental design, contributed to the acquisition, analysis and interpretation of all the experiments, drafted and revised the work. CL, AC, and CG performed the experiments and contributed to acquisition and analysis of most of the results and revised the work. GC, MP, GL, and JV performed part of the experiments and revised the work. AT, LB, J-AF, and JM provided the anticonvulsant, contributed to data interpretation and revised the work.

## Conflict of Interest Statement

AT, GB, and other inventors share a patent on the possible therapeutic use of growth hormone secretagogues for epileptic disorders (patent 0001399610 – 2013; http://www.uibm.gov.it/uibm/dati/Avanzata.aspx?load=info_list_uno&id=1800628&table=Invention&#ancoraSearch). The other authors declare that the research was conducted in the absence of any commercial or financial relationships that could be construed as a potential conflict of interest. The reviewer FW and handling Editor declared their shared affiliation.
